# Body Mass Index and LDL Cholesterol Across Controls, Hypertension, and Ischemic Heart Disease with Preserved Ejection Fraction: A Single-Center Retrospective Cross-Sectional Study

**DOI:** 10.3390/jcm15187096

**Published:** 2026-09-13

**Authors:** Gheorghe Stoichescu-Hogea, Maria-Laura Craciun, Ana-Maria Pah, Cristiana Adina Avram, Maria Rada, Gabriela Otiman, Cornel Jupaneant, Cristina Ples, Abdeldayem Mahmoud, Simona Ruxanda Dragan

**Affiliations:** 1 Department of Cardiology, “Victor Babeș” University of Medicine and Pharmacy of Timișoara, Eftimie Murgu Square 2, 300041 Timișoara, Romania; gheorghe.stoichescu-hogea@umft.ro (G.S.-H.); laura.craciun@umft.ro (M.-L.C.); rada.maria@umft.ro (M.R.); otiman.gabriela@umft.ro (G.O.); simona.dragan@umft.ro (S.R.D.); 2 Department of Internal Medicine I, “Victor Babeș” University of Medicine and Pharmacy of Timișoara, Eftimie Murgu Square 2, 300041 Timișoara, Romania; 3 Doctoral School, “Victor Babeș” University of Medicine and Pharmacy of Timișoara, Eftimie Murgu Square 2, 300041 Timișoara, Romania; jupaneant.cornel@gmail.com (C.J.); cristina.stoichescu-hogea@umft.ro (C.P.); 4 Department of Family Medicine, “Victor Babeș” University of Medicine and Pharmacy of Timișoara, Eftimie Murgu Square 2, 300041 Timișoara, Romania; mahmoodemadsalama@gmail.com

**Keywords:** body mass index, LDL cholesterol, obesity, hypertension, ischemic heart disease, preserved ejection fraction, cardiometabolic phenotype, statin therapy, retrospective study

## Abstract

**Background/Objectives:** We compared BMI and LDL-C across database-defined reference controls, hypertension, and preserved-EF ischemic heart disease without assuming HFpEF. **Methods:** This single-center retrospective cross-sectional study included 128 adults (20 controls, 46 with hypertension, and 62 in the preserved-EF ischemic group). Analyses included nonparametric tests, multinomial regression, clinical-group models with and without statin adjustment, and a post hoc common-age sensitivity analysis. **Results:** Median BMI was 25.89, 28.25, and 29.00 kg/m^2^, respectively (*p* < 0.001); median LDL-C was 86.0, 109.5, and 100.0 mg/dL (*p* = 0.055), alongside statin use of 0%, 50.0%, and 87.1%. Per 5 kg/m^2^ higher BMI, the adjusted RRR for preserved-EF ischemic versus control status was 3.94 (95% CI 1.60–9.71; *p* = 0.003) and remained 3.48 (95% CI 1.35–8.97; *p* = 0.010) among participants aged 40–68 years. In clinical-group models, BMI estimates were similar without and with statin adjustment (OR 1.76 and 1.89); LDL-C was not independently associated with preserved-EF ischemic status. **Conclusions:** BMI differed more consistently across groups than treated, contemporaneous LDL-C. These associations are imprecise and describe group status; they do not establish causation, progression, prospective HF risk, or HFpEF.

## 1. Introduction

Heart failure (HF) is a clinical syndrome of symptoms and/or signs attributable to a structural or functional cardiac abnormality. Diagnosis requires integrated clinical and objective assessment; natriuretic peptides are valuable supportive biomarkers but are not uniformly elevated and must be interpreted alongside evidence of abnormal cardiac structure or function, elevated filling pressures, or congestion [1,2,3]. Contemporary frameworks distinguish individuals at risk for HF, patients with pre-HF structural or biomarker abnormalities, and patients with symptomatic HF. Preserved left ventricular ejection fraction (LVEF) alone is therefore insufficient to establish heart failure with preserved ejection fraction (HFpEF).

HFpEF is a heterogeneous syndrome shaped by aging, hypertension, obesity, diabetes, atrial fibrillation, renal dysfunction, coronary artery disease, and systemic inflammation [4]. The comorbidity-driven paradigm proposes that chronic low-grade inflammation and coronary microvascular endothelial dysfunction impair nitric oxide-cyclic guanosine monophosphate-protein kinase G signaling, promoting cardiomyocyte hypertrophy, titin hypophosphorylation, fibrosis, and increased diastolic stiffness [5]. Data-driven phenomapping further demonstrates that clinically distinct HFpEF subgroups differ in structure, hemodynamics, treatment response, and prognosis [6].

Obesity is a general cardiovascular risk factor, but it is not a single biological phenotype. BMI is a convenient index of body size rather than a direct measure of adiposity; it cannot distinguish fat from lean mass or visceral and ectopic fat from subcutaneous or gluteofemoral fat. Visceral and epicardial adiposity may be especially relevant to insulin resistance, inflammation, pericardial restraint, and adverse cardiac remodeling, so individuals with identical BMI can have different metabolic and cardiac phenotypes [7,8]. Invasive phenotyping supports an obesity-related HFpEF phenotype characterized by greater plasma volume, concentric remodeling, epicardial adiposity, biventricular interaction, higher exercise filling pressures, and impaired pulmonary vascular reserve [7,8]. Obesity and antihypertensive burden are incorporated into the H2FPEF diagnostic score, whereas the HFA-PEFF algorithm requires complementary functional, morphological, and biomarker evidence and recommends exercise or invasive testing when diagnostic probability remains intermediate [9,10].

Recent longitudinal evidence links excess adiposity to incident HF. A 2023 dose–response meta-analysis of 35 prospective studies including more than 1.1 million adults found positive associations for BMI, waist circumference, and waist-to-hip ratio, with stronger pooled associations for HFpEF than HFrEF [11]. In the Women’s Health Initiative, hypertension carried the largest population-attributable risk for HFpEF, followed by obesity [12], while pooled community cohorts showed stronger associations of obesity and several cardiometabolic traits with incident HFpEF than with HF with reduced ejection fraction [13]. LDL-C occupies a complementary position: observed associations with incident HF vary with diabetes, ischemic burden, lipid fraction, and treatment exposure [14,15]. LDL-containing particles remain causally related to atherosclerotic cardiovascular disease (ASCVD), and LDL-C lowering is determined by total cardiovascular risk rather than by a cross-sectional cardiac phenotype [16,17].

The present database offered an opportunity to compare BMI and LDL-C across reference controls, patients with hypertension, and patients with ischemic heart disease and preserved LVEF. The third group was historically labeled ICpFE in the source worksheet; however, the available variables did not permit independent adjudication of HFpEF according to contemporary criteria. We therefore use the term “preserved-EF ischemic heart disease group” for the study cohort; “HFpEF” is reserved for adjudicated syndromes described in the external literature or proposed for future research. The primary objective was to compare BMI and LDL-C across the three groups and estimate their independent associations with cross-sectional group status. We hypothesized that BMI would show a more coherent gradient than LDL-C because measured LDL-C would be strongly modified by unequal statin exposure.

## 2. Materials and Methods

### 2.1. Study Design and Population

This was a single-center, retrospective cross-sectional secondary analysis of a pre-existing, anonymized clinical database from the Institute of Cardiovascular Diseases Timișoara, Romania. The clinical cohorts comprised adults hospitalized and evaluated during 2018–2019 within the institutional research program. The database contained three prespecified worksheets: 62 patients labeled “ICpFE”, 46 patients with hypertension (“HTA”), and 20 controls, yielding 128 participants. It contained no reduced-EF comparison worksheet; therefore, participants with HFrEF could not be added within this fixed secondary-analysis cohort. All available eligible records were included, and no a priori sample-size calculation was performed.

For this analysis, group membership was inherited from the original worksheets and operationally characterized from the recorded diagnoses and LVEF. The control group is best regarded as a database-defined reference group: its records lacked documented hypertension, diabetes mellitus, or clinical cardiac disease, but the available database did not establish health through prospective standardized screening. The hypertension group comprised patients with essential hypertension and preserved LVEF. The third worksheet predominantly contained patients with coronary or ischemic heart disease and LVEF >= 50%; it is therefore described throughout as the preserved-EF ischemic heart disease group rather than HFpEF. Records required a valid group assignment and an available LDL-C value. The report follows STROBE recommendations for cross-sectional observational studies [18].

### 2.2. Variables, Group Harmonization, and Data Quality

Prespecified variables were age, sex, BMI, LDL-C, hypertension grade, diabetes mellitus, LVEF, and statin therapy. Self-identified race and ethnicity were not recorded in the source database and could not be analyzed. BMI was analyzed continuously and dichotomized at 30 kg/m^2^ to describe obesity. LDL-C was analyzed continuously and descriptively dichotomized at 100 mg/dL. This threshold was used for sample description and was not intended as a universal treatment target because recommended LDL-C goals depend on total cardiovascular risk, ASCVD status, and treatment context [17].

Column labels and coding were harmonized across worksheets before analysis. Sex was coded as male or female, and binary clinical variables as present or absent. Direct identifiers were not retained in the analytical dataset. One control-group BMI value recorded as 44,309 kg/m^2^ was biologically implausible and treated as a data-entry error; it was set to missing only for BMI-dependent analyses, while the participant remained in analyses not requiring BMI. No LDL-C observations were excluded, no statistical imputation was applied, and all model-specific sample sizes are reported.

### 2.3. Outcomes and Interpretation of Cross-Sectional Group Comparisons

The primary analytical outcome was membership in one of the three source-defined groups. Multinomial logistic regression used controls as the reference category and estimated relative risk ratios (RRRs), the conventional effect measure from this model, for hypertension and preserved-EF ischemic group membership. In this cross-sectional setting, these RRRs compare modeled group probabilities and are not prospective disease-risk estimates. A secondary binary model restricted to the two clinical groups estimated odds ratios (ORs) for preserved-EF ischemic versus hypertension status.

These outcomes are cross-sectional group classifications, not incident HF events or observed disease transitions. Consequently, expressions such as “HF-related phenotype” or “cardiovascular continuum” refer only to phenotypic associations with the recorded groups. The database did not contain prospective follow-up, HF hospitalization, natriuretic peptides, complete diastolic echocardiographic indices, objective congestion measures, or exercise hemodynamics. No validated HF risk score was calculated, no contemporary HFpEF diagnosis was reconstructed, and no temporal or prognostic inference was attempted.

### 2.4. Statistical Analysis

Continuous variables were inspected for distributional shape and are presented as median and interquartile range (IQR). Given the modest sample size and non-normal distributions, three-group comparisons used the Kruskal–Wallis test. Pairwise Mann–Whitney U tests were performed when informative and adjusted using the Holm method. Categorical variables are reported as *n/N* (%) and were compared using chi-square tests. Effect estimates, confidence intervals, and multiplicity-adjusted results were emphasized rather than isolated nominal *p*-values.

Spearman rank correlations quantified the association between BMI and LDL-C in the pooled population and within each group; the four correlation *p*-values were Holm-adjusted. The primary multinomial model included LDL-C per 10 mg/dL, BMI per 5 kg/m^2^, age per 10 years, and male sex. Statin therapy was not added to this three-group model because no controls received statins, creating structural separation. Among the 108 participants in the two clinical groups, Model A included the same exposures plus diabetes mellitus and hypertension grade, and Model B additionally included statin therapy. Presenting both models allowed direct assessment of how statin adjustment influenced the BMI and LDL-C estimates.

To assess treatment-related distortion of observed LDL-C, an exploratory sensitivity analysis compared LDL-C distributions after stratification by statin use. Because only eight statin-naive participants belonged to the preserved-EF ischemic group, those results were interpreted descriptively. A post hoc age-comparability analysis then restricted all three groups to their common observed age support (40–68 years) and repeated the primary multinomial model without changing covariates. This restriction reduced the age imbalance without reassigning or matching participants.

Effect estimates are reported with 95% confidence intervals (CIs). Statistical tests were two-sided, with *p* < 0.05 considered statistically significant. Analyses were performed using Python 3.13, NumPy 2.3.5, SciPy 1.17.0, and statsmodels 0.14.6. Multivariable analyses used complete cases for included covariates.

### 2.5. Ethics

This study was conducted in accordance with the Declaration of Helsinki and approved by the Research-Development Ethics Committee of the Institute of Cardiovascular Diseases Timișoara (approval no. 1432, 20 February 2019). The present work is a secondary analysis of the approved retrospective study dataset.

Written informed consent, including consent for the use of anonymized clinical and laboratory data for research purposes, was obtained from all participants at the time of hospital admission, in accordance with the institutional ethics approval.

## 3. Results

### 3.1. Cohort Characteristics and Data Completeness

The analysis included 128 participants: 20 controls, 46 patients with hypertension, and 62 patients in the preserved-EF ischemic heart disease group. LDL-C data were available for all participants. BMI was available for 127 participants after the single implausible control value was set to missing. The clinical groups were older than controls, whereas sex distribution did not differ significantly across groups.

Statin therapy showed a marked group gradient: 0% in controls, 50.0% in hypertension, and 87.1% in the preserved-EF ischemic group (*p* < 0.001). This imbalance was clinically expected but central to interpreting LDL-C. Diabetes was absent in controls and present in 34.8% of the hypertension group and 24.2% of the preserved-EF ischemic group (*p* = 0.010). Median LVEF was 55% in all three groups and did not differ significantly (*p* = 0.262; Table 1).

### 3.2. BMI Across the Three Clinical Groups

**Figure 1:**
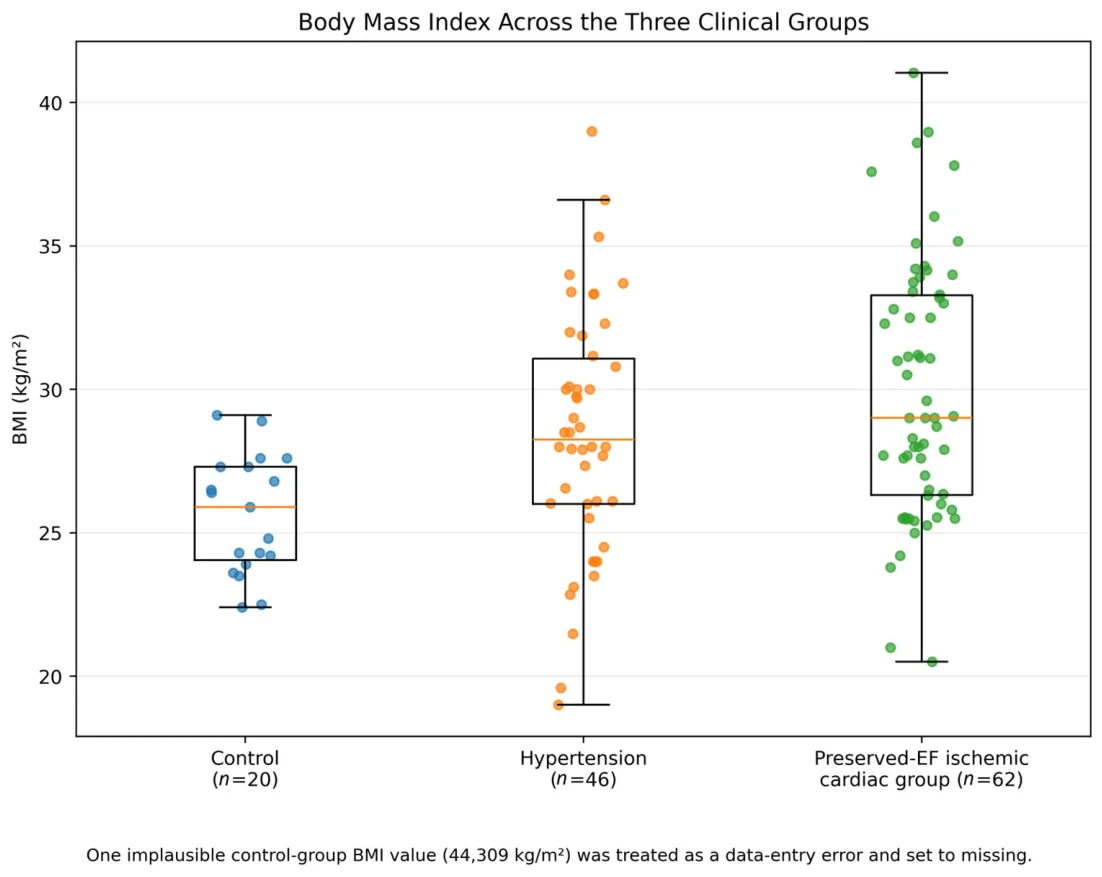
BMI distribution across controls, hypertension, and the preserved-EF ischemic heart disease group. Boxes indicate the interquartile range, horizontal lines indicate medians, whiskers represent the conventional boxplot range, and points show individual observations. One implausible control BMI value was set to missing.

Median BMI differed across groups (Kruskal–Wallis *p* < 0.001), with values of 25.89 kg/m^2^ (IQR 24.05–27.30) in controls, 28.25 kg/m^2^ (IQR 26.01–31.08) in hypertension, and 29.00 kg/m^2^ (IQR 26.31–33.27) in the preserved-EF ischemic group (Figure 1). After Holm correction, BMI was higher in hypertension versus controls (adjusted *p* = 0.008) and in the preserved-EF ischemic group versus controls (adjusted *p* < 0.001). The difference between the two clinical groups was not significant after correction (adjusted *p* = 0.158).

Obesity was absent among the 19 controls with valid BMI measurements, compared with 37.0% of the hypertension group and 45.2% of the preserved-EF ischemic group (*p* = 0.001). Thus, the strongest unadjusted separation occurred between controls and either clinical group, while differentiation between the two clinical populations required multivariable assessment.

### 3.3. LDL-C, Statin Exposure, and Group Status

**Figure 2:**
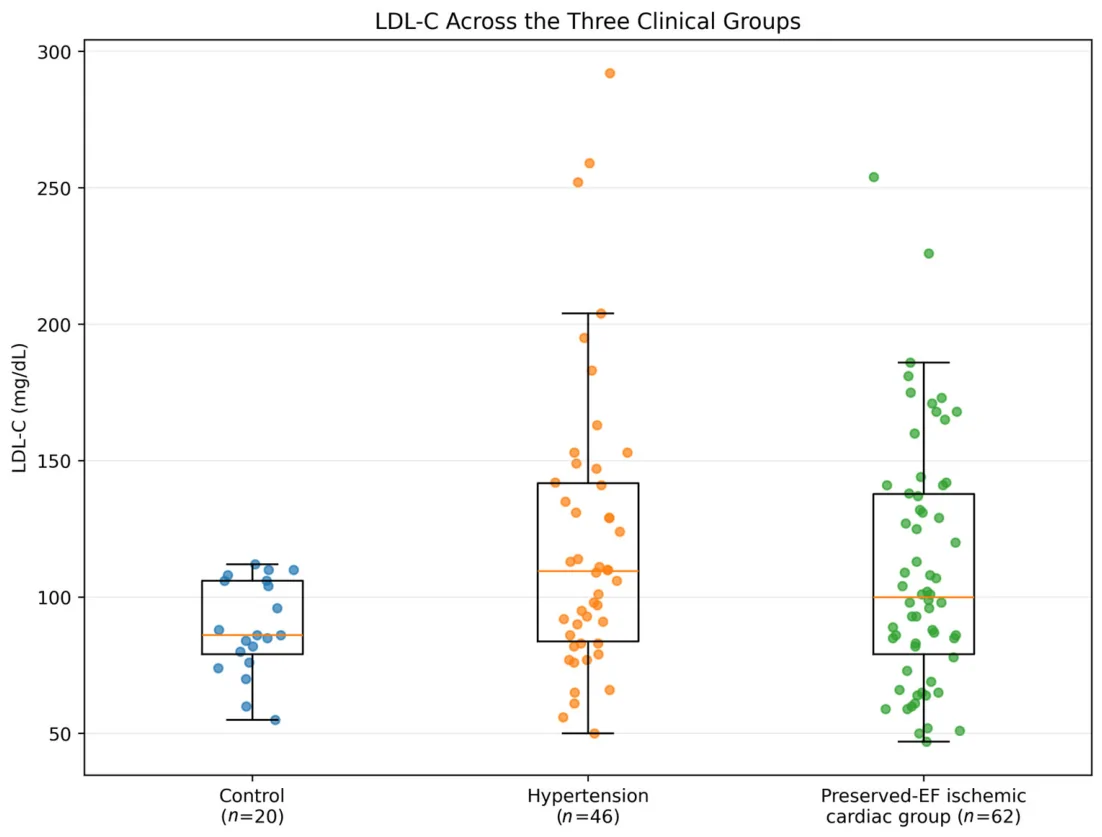
LDL-C distribution across the three groups, with individual observations superimposed on boxplots. LDL-C, low-density lipoprotein cholesterol.

Median LDL-C was 86.0 mg/dL (IQR 79.0–106.0) in controls, 109.5 mg/dL (IQR 83.8–141.8) in hypertension, and 100.0 mg/dL (IQR 79.0–137.8) in the preserved-EF ischemic group (overall *p* = 0.055; Figure 2). In pairwise analyses, LDL-C was higher in hypertension than controls after Holm correction (adjusted *p* = 0.033). Neither the preserved-EF ischemic versus control comparison (adjusted *p* = 0.222) nor the preserved-EF ischemic versus hypertension comparison (adjusted *p* = 0.334) was significant.

The proportion with LDL-C >= 100 mg/dL was 35.0% in controls, 56.5% in hypertension, and 50.0% in the preserved-EF ischemic group (*p* = 0.275). The lower median LDL-C in the preserved-EF ischemic group than in hypertension occurred despite more extensive ischemic disease and coincided with substantially greater statin exposure, indicating that raw LDL-C values cannot be interpreted independently of treatment context.

In the exploratory statin-stratified analysis, LDL-C differed across statin-naive groups (*p* = 0.008): median 86.0 mg/dL (IQR 79.0–106.0; *n* = 20) in controls, 110.0 mg/dL (IQR 84.5–135.5; *n* = 23) in hypertension, and 130.5 mg/dL (IQR 119.25–146.0; *n* = 8) in the preserved-EF ischemic group. After Holm correction, the preserved-EF ischemic versus control comparison remained significant (adjusted *p* = 0.018), while hypertension versus control was borderline (adjusted *p* = 0.052). Among statin-treated participants, LDL-C did not differ between hypertension (101.0 mg/dL, IQR 87.5–144.0; *n* = 23) and the preserved-EF ischemic group (98.0 mg/dL, IQR 74.25–136.25; *n* = 54; *p* = 0.238). These analyses support treatment-related attenuation of the crude LDL-C gradient but are limited by the small statin-naive ischemic subgroup.

### 3.4. Correlations Between BMI and LDL-C

BMI and LDL-C were not significantly correlated in the pooled cohort (Spearman rho = 0.099, unadjusted *p* = 0.267; Holm-adjusted *p* = 0.800). The nominal control-group association (rho = 0.460, *p* = 0.047) did not remain significant after correction (adjusted *p* = 0.190). Correlations were negligible in hypertension (rho = 0.022) and the preserved-EF ischemic group (rho = 0.011), both adjusted *p* = 1.000. LDL-C therefore did not function as a surrogate for adiposity in this dataset. LDL-C therefore did not function as a surrogate for adiposity in this dataset (Table 2).

### 3.5. Adjusted Multinomial Associations with Clinical Group Status

The multinomial model included 127 complete cases and was statistically significant overall (likelihood-ratio *p* < 0.001; pseudo-R^2^ = 0.159). For hypertension versus control status, the adjusted RRR per 10 mg/dL higher LDL-C was 1.30 (95% CI 1.04–1.62; *p* = 0.023), and the RRR per 10-year age increment was 2.09 (95% CI 1.24–3.51; *p* = 0.006). The BMI estimate was imprecise and did not reach statistical significance (RRR 2.41 per 5 kg/m^2^, 95% CI 0.98–5.93; *p* = 0.054).

For preserved-EF ischemic versus control status, higher BMI and older age were independently associated with group membership. Each 5 kg/m^2^ higher BMI corresponded to an adjusted RRR of 3.94 (95% CI 1.60–9.71; *p* = 0.003), and each 10-year age increment to an RRR of 2.35 (95% CI 1.38–3.99; *p* = 0.002). LDL-C was not independently associated with group status (RRR 1.22 per 10 mg/dL, 95% CI 0.98–1.53; *p* = 0.079). The wide BMI CI indicates substantial uncertainty about the magnitude of the association; sex was not significant in either comparison (Table 3).

### 3.6. Preserved-EF Ischemic Group Versus Hypertension

Among the 108 participants in the two clinical groups, Model A omitted statin therapy. Each 5 kg/m^2^ higher BMI was associated with preserved-EF ischemic rather than hypertension status (adjusted OR 1.76, 95% CI 1.07–2.90; *p* = 0.027), whereas LDL-C was not associated with group status (OR 0.94 per 10 mg/dL, 95% CI 0.86–1.02; *p* = 0.154). In Model B, which added statin therapy, the corresponding BMI estimate was similar (OR 1.89, 95% CI 1.09–3.28; *p* = 0.023), and LDL-C remained non-significant (OR 0.96, 95% CI 0.87–1.05; *p* = 0.368).

**Figure 3:**
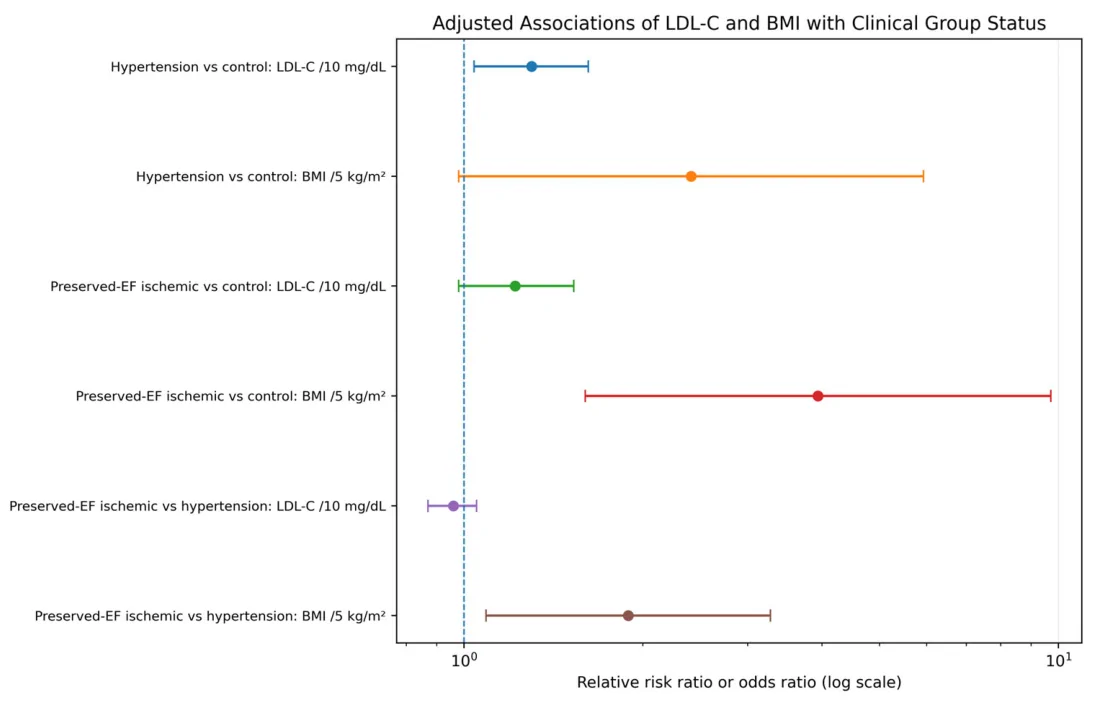
Adjusted associations of LDL-C and BMI with clinical group status. Hypertension and preserved-EF ischemic versus control estimates are RRRs from the multinomial model; preserved-EF ischemic versus hypertension estimates are ORs from Model B, which includes statin therapy. Bars indicate 95% CIs; the *x*-axis is logarithmic.

In Model B, statin therapy was strongly associated with preserved-EF ischemic group membership (OR 7.98, 95% CI 2.70–23.59; *p* < 0.001). This estimate is best interpreted as confounding by indication and a marker of established ischemic disease treatment, not a causal adverse effect. Age, sex, diabetes, and hypertension grade were not independently significant in either model. The similar BMI estimates and null LDL-C estimates before and after statin adjustment indicate that these conclusions were not created by inclusion of the statin covariate (Table 4; Figure 3).

### 3.7. Age-Comparable Sensitivity Analysis

The common observed age range across all three groups was 40–68 years, yielding 85 participants: 16 controls, 29 with hypertension, and 40 in the preserved-EF ischemic group. Median ages were 57.0, 59.0, and 61.0 years, respectively, and no longer differed significantly (Kruskal–Wallis *p* = 0.382).

Within this age-comparable subset, the association of BMI with preserved-EF ischemic versus control status remained present (RRR 3.48 per 5 kg/m^2^, 95% CI 1.35–8.97; *p* = 0.010), whereas LDL-C was not significant (RRR 1.20 per 10 mg/dL, 95% CI 0.96–1.50; *p* = 0.103). For hypertension versus control status, LDL-C remained associated with group membership (RRR 1.28, 95% CI 1.02–1.60; *p* = 0.034), while the BMI estimate remained imprecise (RRR 2.32, 95% CI 0.88–6.07; *p* = 0.088). The subset included only 16 controls, so the analysis supports the direction rather than the precise magnitude of the primary BMI association (Table 5).

## 4. Discussion

### 4.1. Principal Findings

This single-center analysis found that BMI differed more consistently across database-defined groups than contemporaneous LDL-C. Higher BMI was associated with preserved-EF ischemic group membership versus controls and hypertension, but the CIs were wide. The BMI association versus controls remained in the common-age subset, and the disease-group BMI estimate was similar before and after statin adjustment. LDL-C followed a non-monotonic distribution, coincided with unequal statin exposure, and was not independently associated with preserved-EF ischemic status. BMI and LDL-C were not robustly correlated. These are cross-sectional phenotype comparisons, not estimates of prediction, progression, or causation.

The third cohort was not adjudicated as HFpEF: preserved LVEF and ischemic disease do not establish the syndrome without appropriate symptoms, objective cardiac or hemodynamic evidence, and integrated diagnostic assessment [1,2,3,4,9,10]. Likewise, the control worksheet was a reference group without recorded target diagnoses, not a cohort verified as healthy by standardized screening. Because the database also contained no reduced-EF group, the results cannot be extended to HFrEF or used to compare HF phenotypes.

### 4.2. BMI, Age, and Phenotypic Interpretation

Age was an important source of imbalance because the control group was younger. Multivariable age adjustment was therefore supplemented by restriction to the common 40–68-year age range. In that subset, median age no longer differed significantly and the BMI association for preserved-EF ischemic versus control status remained present (RRR 3.48), although its 95% CI (1.35–8.97) was still wide. This concordance supports robustness of the direction of association, not a precise effect magnitude; residual confounding and instability from only 16 age-comparable controls remain possible.

Hypertension and obesity are established correlates of incident HFpEF in longitudinal cohorts [11,12,13,19], and ischemic disease can add further structural and metabolic burden. Nevertheless, the present groups are not stages in a deterministic continuum. The observed association indicates that overall body size differed after covariate adjustment; it does not show that hypertension progressed to ischemic disease or HFpEF, nor that BMI mediated such a transition.

### 4.3. BMI, Fat Distribution, and Biological Plausibility

Excess adiposity can increase blood volume, ventricular filling requirements, sodium retention, inflammation, and neurohormonal activation. Visceral and epicardial fat may also promote pericardial restraint, ventricular interaction, coronary microvascular dysfunction, and higher exercise filling pressures [5,7,8]. These mechanisms make the BMI pattern biologically plausible but cannot identify which adipose depot, if any, accounted for the association in this dataset.

BMI also misses sleep apnea, insulin resistance, skeletal–muscle dysfunction, pulmonary limitation, physical inactivity, and the comparatively lower natriuretic peptide concentrations sometimes observed in obesity [7,8,9,10]. Brief interventional evidence from adjudicated obesity-related HFpEF populations shows that exercise, caloric restriction, semaglutide, and tirzepatide can improve selected clinical outcomes [20,21,22,23,24], but these trials neither validate HFpEF in the present cohort nor convert a cross-sectional BMI association into a treatment effect. Associations between BMI and outcomes after established HF are a separate question [25].

### 4.4. BMI, LDL-C, and Regional Adiposity

The absent BMI-LDL-C correlation is biologically plausible because obesity-related dyslipidemia often involves triglyceride-rich remnants, lower HDL-C, and small dense LDL particles rather than a proportional rise in LDL-C [26]. Medication exposure, diabetes, diet, renal function, and clinical selection can further weaken a simple monotonic relationship. BMI and LDL-C should therefore be treated as complementary rather than interchangeable measurements.

BMI cannot distinguish fat from lean mass or visceral and ectopic fat from subcutaneous and gluteofemoral fat [27,28,29]. This matters because abdominal adiposity is associated with insulin resistance and a less favorable inflammatory profile, whereas lower-body adipose tissue may provide comparatively favorable metabolic storage [30]. Waist-to-hip ratio (WHR) can distinguish android from gynoid patterns obscured by similar BMI; in healthy women with obesity, WHR-defined phenotypes were associated with different left-ventricular deformation despite comparable BMI [31]. The absence of waist and hip measurements is therefore a central limitation, and future studies should test whether WHR adds discrimination beyond BMI.

### 4.5. LDL-C and Statin Context

LDL-C was highest in hypertension and intermediate in the preserved-EF ischemic group, where 87.1% received statins. In the disease-group analysis, the BMI estimate changed a little after adding statin therapy (OR 1.76 to 1.89), while LDL-C remained non-significant (OR 0.94 to 0.96). The strong statin coefficient in the adjusted model is expected from treatment indication and ischemic disease burden; it should not be interpreted as a harmful statin effect.

The stratified analysis likewise showed the highest LDL-C in the small statin-naive preserved-EF ischemic subgroup but no difference between treated clinical groups. Thus, a single current LDL-C value cannot recover untreated or lifetime exposure. LDL-containing particles remain causal for ASCVD, and treatment should follow documented ASCVD, total risk, pretreatment values when available, treatment intensity, and response rather than these cross-sectional comparisons [16,17,32,33,34].

### 4.6. Clinical and Research Implications

Clinically, BMI is inexpensive but should be complemented by waist and hip circumference, WHR, weight trajectory, blood-pressure and glycemic burden, and evidence of congestion or functional limitation. In a symptomatic person with preserved LVEF, obesity and hypertension may increase suspicion but cannot establish HFpEF. LDL-C should be interpreted in relation to treatment, pretreatment concentration, and total ASCVD risk; neither the absent BMI-LDL-C correlation nor the lower treated LDL-C in the ischemic group supports treatment de-escalation.

Future prospective work should recruit sufficiently sized and age-comparable reference, hypertension, ischemic heart disease, HFrEF, and independently adjudicated HFpEF cohorts. It should record race and ethnicity, waist and hip circumference, body composition, detailed metabolic and lipid profiles, standardized echocardiography, natriuretic peptides, congestion measures, exercise phenotyping when indicated, medication intensity and adherence, and longitudinal cardiovascular outcomes. Incremental analyses should test whether WHR or direct visceral-fat measures improve discrimination beyond BMI.

### 4.7. Strengths and Limitations

The strengths include complete LDL-C data, near-complete BMI data, transparent handling of an implausible value, multiplicity-adjusted analyses, explicit separation of the historical source label from contemporary HFpEF, direct comparison of models with and without statin adjustment, and an age-common-support sensitivity analysis.

The principal limitations are the retrospective cross-sectional design and modest sample, particularly the 20-person control group and eight statin-naive ischemic participants. Wide CIs limit precision, and the age-restricted analysis retained only 16 controls. BMI could not distinguish visceral from subcutaneous fat, abdominal from gluteofemoral distribution, or fat from lean mass; waist circumference, hip circumference, WHR, and body-composition measures were unavailable.

Group classification was inherited from source worksheets, screening logs could not be fully reconstructed, and the reference controls were not independently verified as healthy. Preserved LVEF, ischemic disease, and NYHA class did not establish HFpEF, and no HFrEF comparator was available. Race and ethnicity were not recorded, limiting generalizability and precluding assessments of race-related heterogeneity. Residual age confounding may persist despite adjustment and restriction. Natriuretic peptides, complete diastolic assessment, congestion, pretreatment LDL-C, statin intensity and adherence, several lifestyle and clinical confounders, and longitudinal outcomes were also unavailable. The exploratory models were not internally or externally validated and must not be interpreted as causal or prospective HF-risk estimates.

## 5. Conclusions

In this 128-person retrospective cross-sectional dataset, BMI differed across reference controls, hypertension, and preserved-EF ischemic heart disease more consistently than contemporaneous LDL-C. The adjusted BMI association with preserved-EF ischemic group status was directionally consistent in the common-age subset and in clinical-group models with and without statin adjustment, but wide CIs and the small control group limit precision. LDL-C was non-monotonic across groups and strongly contextualized by unequal statin exposure. These findings describe source-defined group differences only; they do not establish HFpEF, HFrEF comparisons, temporal progression, causation, prognosis, or future HF risk. Confirmation requires larger prospective cohorts with reduced-EF and adjudicated preserved-EF groups, regional adiposity measures, and longitudinal outcomes.

## Figures and Tables

**Table 1:** Clinical characteristics across the three groups.

*p*-Value	Preserved-EF Ischemic Heart Disease (*n* = 62)	Hypertension (*n* = 46)	Control (*n* = 20)	Characteristic
<0.001	63.0 (60.0–69.0)	62.5 (53.5–70.8)	50.5 (42.0–59.8)	Age, years
0.231	42/62 (67.7%)	24/46 (52.2%)	11/20 (55.0%)	Male sex
<0.001	29.00 (26.31–33.27)	28.25 (26.01–31.08)	25.89 (24.05–27.30)	BMI, kg/m^2^
0.001	28/62 (45.2%)	17/46 (37.0%)	0/19 (0.0%)	Obesity (BMI ≥ 30 kg/m^2^)
0.055	100.0 (79.0–137.8)	109.5 (83.8–141.8)	86.0 (79.0–106.0)	LDL-C, mg/dL
0.275	31/62 (50.0%)	26/46 (56.5%)	7/20 (35.0%)	LDL-C ≥ 100 mg/dL
0.010	15/62 (24.2%)	16/46 (34.8%)	0/20 (0.0%)	Diabetes mellitus
<0.001	54/62 (87.1%)	23/46 (50.0%)	0/20 (0.0%)	Statin therapy
0.262	55.0 (50.0–55.0)	55.0 (50.0–60.0)	55.0 (55.0–55.0)	LVEF, %

Values are median (IQR) or n/N (%). Continuous variables were compared using Kruskal–Wallis tests; categorical variables used chi-square tests. The obesity denominator in controls is 19 because one implausible BMI entry was set to missing. BMI, body mass index; LDL-C, low-density lipoprotein cholesterol; LVEF, left ventricular ejection fraction.

**Table 2:** Spearman correlations between BMI and LDL-C.

Holm-Adjusted *p*	Unadjusted *p*	Spearman ρ	*n*	Population
0.800	0.267	0.099	127	Pooled
0.190	0.047	0.460	19	Control
1.000	0.883	0.022	46	Hypertension
1.000	0.933	0.011	62	Preserved-EF ischemic group

Holm adjustment was applied across the four correlation analyses.

**Table 3:** Adjusted multinomial logistic regression with controls as the reference group.

*p*-Value	95% CI	Adjusted RRR	Predictor	Comparison
0.023	1.04–1.62	1.30	LDL-C per 10 mg/dL	Hypertension vs. control
0.054	0.98–5.93	2.41	BMI per 5 kg/m^2^	Hypertension vs. control
0.006	1.24–3.51	2.09	Age per 10 years	Hypertension vs. control
0.967	0.28–3.40	0.97	Male sex	Hypertension vs. control
0.079	0.98–1.53	1.22	LDL-C per 10 mg/dL	Preserved-EF ischemic vs. control
0.003	1.60–9.71	3.94	BMI per 5 kg/m^2^	Preserved-EF ischemic vs. control
0.002	1.38–3.99	2.35	Age per 10 years	Preserved-EF ischemic vs. control
0.205	0.64–7.98	2.26	Male sex	Preserved-EF ischemic vs. control

*n* = 127. The model included LDL-C per 10 mg/dL, BMI per 5 kg/m^2^, age per 10 years, and male sex. RRRs describe cross-sectional group membership and are not prospective HF risk ratios.

**Table 4:** Comparative adjusted logistic models for preserved-EF ischemic heart disease versus hypertension status.

*p*-Value	95% CI	Adjusted OR	Predictor
			Model A: without statin therapy
0.154	0.86–1.02	0.94	LDL-C per 10 mg/dL
0.027	1.07–2.90	1.76	BMI per 5 kg/m^2^
0.271	0.84–1.86	1.25	Age per 10 years
0.058	0.97–5.45	2.30	Male sex
0.172	0.20–1.33	0.52	Diabetes mellitus
0.333	0.46–1.30	0.78	Hypertension grade
			Model B: with statin therapy
0.368	0.87–1.05	0.96	LDL-C per 10 mg/dL
0.023	1.09–3.28	1.89	BMI per 5 kg/m^2^
0.185	0.86–2.16	1.36	Age per 10 years
0.386	0.59–3.99	1.53	Male sex
0.157	0.17–1.33	0.48	Diabetes mellitus
0.098	0.33–1.10	0.60	Hypertension grade
<0.001	2.70–23.59	7.98	Statin therapy

*n* = 108 in both models. Model A included LDL-C, BMI, age, male sex, diabetes mellitus, and hypertension grade; Model B additionally included statin therapy. ORs describe cross-sectional group membership. The statin estimate is highly susceptible to confounding by indication.

**Table 5:** Age-comparable sensitivity analysis: adjusted multinomial logistic regression with controls as the reference group.

*p*-Value	95% CI	Adjusted RRR	Predictor	Comparison
0.034	1.02–1.60	1.28	LDL-C per 10 mg/dL	Hypertension vs. control
0.088	0.88–6.07	2.32	BMI per 5 kg/m^2^	Hypertension vs. control
0.360	0.64–3.47	1.49	Age per 10 years	Hypertension vs. control
0.893	0.23–3.56	0.91	Male sex	Hypertension vs. control
0.103	0.96–1.50	1.20	LDL-C per 10 mg/dL	Preserved-EF ischemic vs. control
0.010	1.35–8.97	3.48	BMI per 5 kg/m^2^	Preserved-EF ischemic vs. control
0.103	0.87–4.84	2.05	Age per 10 years	Preserved-EF ischemic vs. control
0.396	0.47–6.83	1.79	Male sex	Preserved-EF ischemic vs. control

*n* = 85 participants aged 40–68 years. The model included LDL-C per 10 mg/dL, BMI per 5 kg/m^2^, age per 10 years, and male sex. RRRs describe cross-sectional group membership and are not prospective HF risk ratios.

## Data Availability

De-identified clinical and laboratory data supporting the findings of this study are available from the corresponding author upon reasonable request, subject to institutional data-sharing policies.

## Abbreviations

The following abbreviations are used in this manuscript:

ASCVD	atherosclerotic cardiovascular disease
BMI	body mass index
CI	confidence interval
HF	heart failure
HFpEF	heart failure with preserved ejection fraction
ICpFE	historical source-worksheet label for the preserved-EF ischemic cohort; not an adjudicated HFpEF diagnosis
IQR	interquartile range
LDL-C	low-density lipoprotein cholesterol
LVEF	left ventricular ejection fraction
NYHA	New York Heart Association
OR	odds ratio
RRR	relative risk ratio
WHR	waist-to-hip ratio

## References

[1] Bozkurt B., Coats A.J.S., Tsutsui H., Abdelhamid C.M., Adamopoulos S., Albert N., Anker S.D., Atherton J., Böhm M., Butler J. (2021). Universal Definition and Classification of Heart Failure: A Report of the Heart Failure Society of America, Heart Failure Association of the European Society of Cardiology, Japanese Heart Failure Society and Writing Committee of the Universal Definition of Heart Failure. J. Card. Fail..

[2] McDonagh T.A., Metra M., Adamo M., Gardner R.S., Baumbach A., Böhm M., Burri H., Butler J., Celutkiene J., Chioncel O. (2021). 2021 ESC Guidelines for the Diagnosis and Treatment of Acute and Chronic Heart Failure. Eur. Heart J..

[3] Heidenreich P.A., Bozkurt B., Aguilar D., Allen L.A., Byun J.J., Colvin M.M., Deswal A., Drazner M.H., Dunlay S.M., Evers L.R. (2022). 2022 AHA/ACC/HFSA Guideline for the Management of Heart Failure. Circulation.

[4] Redfield M.M., Borlaug B.A. (2023). Heart Failure with Preserved Ejection Fraction: A Review. JAMA.

[5] Paulus W.J., Tschöpe C. (2013). A Novel Paradigm for Heart Failure with Preserved Ejection Fraction: Comorbidities Drive Myocardial Dysfunction and Remodeling through Coronary Microvascular Endothelial Inflammation. J. Am. Coll. Cardiol..

[6] Shah S.J., Katz D.H., Selvaraj S., Burke M.A., Yancy C.W., Gheorghiade M., Bonow R.O., Huang C.C., Deo R.C. (2015). Phenomapping for Novel Classification of Heart Failure with Preserved Ejection Fraction. Circulation.

[7] Obokata M., Reddy Y.N.V., Pislaru S.V., Melenovsky V., Borlaug B.A. (2017). Evidence Supporting the Existence of a Distinct Obese Phenotype of Heart Failure with Preserved Ejection Fraction. Circulation.

[8] Borlaug B.A., Jensen M.D., Kitzman D.W., Lam C.S.P., Obokata M., Rider O.J. (2022). Obesity and Heart Failure with Preserved Ejection Fraction: New Insights and Pathophysiological Targets. Cardiovasc. Res..

[9] Reddy Y.N.V., Carter R.E., Obokata M., Redfield M.M., Borlaug B.A. (2018). A Simple, Evidence-Based Approach to Help Guide Diagnosis of Heart Failure with Preserved Ejection Fraction. Circulation.

[10] Pieske B., Tschöpe C., de Boer R.A., Fraser A.G., Anker S.D., Donal E., Edelmann F., Fu M., Guazzi M., Lam C.S.P. (2019). How to Diagnose Heart Failure with Preserved Ejection Fraction: The HFA–PEFF Diagnostic Algorithm. Eur. Heart J..

[11] Oguntade A.S., Islam N., Malouf R., Taylor H., Jin D., Lewington S., Lacey B. (2023). Body Composition and Risk of Incident Heart Failure in 1 Million Adults: A Systematic Review and Dose-Response Meta-Analysis of Prospective Cohort Studies. J. Am. Heart Assoc..

[12] Eaton C.B., Pettinger M., Rossouw J., Martin L.W., Foraker R., Quddus A., Liu S., Wampler N.S., Hank Wu W.C., Manson J.E. (2016). Risk Factors for Incident Hospitalized Heart Failure with Preserved versus Reduced Ejection Fraction in a Multiracial Cohort of Postmenopausal Women. Circ. Heart Fail..

[13] Savji N., Meijers W.C., Bartz T.M., Bhambhani V., Cushman M., Nayor M., Kizer J.R., Sarma A., Blaha M.J., Kop W.J. (2018). The Association of Obesity and Cardiometabolic Traits with Incident HFpEF and HFrEF. JACC Heart Fail..

[14] Velagaleti R.S., Massaro J., Vasan R.S., Robins S.J., Kannel W.B., Levy D. (2009). Relations of Lipid Concentrations to Heart Failure Incidence: The Framingham Heart Study. Circulation.

[15] Ebong I.A., Goff D.C., Rodriguez C.J., Chen H., Sibley C.T., Bertoni A.G. (2013). Association of Lipids with Incident Heart Failure among Adults with and without Diabetes Mellitus: Multi-Ethnic Study of Atherosclerosis. Circ. Heart Fail..

[16] Ference B.A., Ginsberg H.N., Graham I., Ray K.K., Packard C.J., Bruckert E., Hegele R.A., Krauss R.M., Raal F.J., Schunkert H. (2017). Low-Density Lipoproteins Cause Atherosclerotic Cardiovascular Disease. 1. Evidence from Genetic, Epidemiologic, and Clinical Studies. Eur. Heart J..

[17] Mach F., Baigent C., Catapano A.L., Koskinas K.C., Casula M., Badimon L., Chapman M.J., De Backer G.G., Delgado V., Ference B.A. (2020). 2019 ESC/EAS Guidelines for the Management of Dyslipidaemias: Lipid Modification to Reduce Cardiovascular Risk. Eur. Heart J..

[18] von Elm E., Altman D.G., Egger M., Pocock S.J., Gøtzsche P.C., Vandenbroucke J.P. (2007). The Strengthening the Reporting of Observational Studies in Epidemiology (STROBE) Statement: Guidelines for Reporting Observational Studies. Lancet.

[19] Ho J.E., Lyass A., Lee D.S., Vasan R.S., Kannel W.B., Larson M.G., Levy D. (2013). Predictors of New-Onset Heart Failure: Differences in Preserved versus Reduced Ejection Fraction. Circ. Heart Fail..

[20] Kitzman D.W., Brubaker P., Morgan T., Haykowsky M., Hundley G., Kraus W.E., Eggebeen J., Nicklas B.J. (2016). Effect of Caloric Restriction or Aerobic Exercise Training on Peak Oxygen Consumption and Quality of Life in Obese Older Patients with Heart Failure with Preserved Ejection Fraction: A Randomized Clinical Trial. JAMA.

[21] Powell-Wiley T.M., Poirier P., Burke L.E., Després J.P., Gordon-Larsen P., Lavie C.J., Lear S.A., Ndumele C.E., Neeland I.J., Sanders P. (2021). Obesity and Cardiovascular Disease: A Scientific Statement from the American Heart Association. Circulation.

[22] Kosiborod M.N., Abildstrøm S.Z., Borlaug B.A., Butler J., Rasmussen S., Davies M., Hovingh G.K., Kitzman D.W., Lindegaard M.L., Møller D.V. (2023). Semaglutide in Patients with Heart Failure with Preserved Ejection Fraction and Obesity. N. Engl. J. Med..

[23] Kosiborod M.N., Petrie M.C., Borlaug B.A., Butler J., Davies M.J., Hovingh G.K., Kitzman D.W., Møller D.V., Shah S.J., Rasmussen S. (2024). Semaglutide in Patients with Obesity-Related Heart Failure and Type 2 Diabetes. N. Engl. J. Med..

[24] Packer M., Zile M.R., Kramer C.M., Baum S.J., Litwin S.E., Menon V., Ge J., Weerakkody G.J., Ou Y., Bunck M.C. (2025). Tirzepatide for Heart Failure with Preserved Ejection Fraction and Obesity. N. Engl. J. Med..

[25] Li S., Zheng Y., Huang Y., He W., Liu X., Zhu W. (2022). Association of Body Mass Index and Prognosis in Patients with HFpEF: A Dose–Response Meta-Analysis. Int. J. Cardiol..

[26] Klop B., Elte J.W.F., Cabezas M.C. (2013). Dyslipidemia in Obesity: Mechanisms and Potential Targets. Nutrients.

[27] Neeland I.J., Ross R., Després J.P., Matsuzawa Y., Yamashita S., Shai I., Seidell J., Magni P., Santos R.D., Arsenault B. (2019). Visceral and Ectopic Fat, Atherosclerosis, and Cardiometabolic Disease: A Position Statement. Lancet Diabetes Endocrinol..

[28] Ashwell M., Gunn P., Gibson S. (2012). Waist-to-Height Ratio Is a Better Screening Tool than Waist Circumference and BMI for Adult Cardiometabolic Risk Factors: Systematic Review and Meta-Analysis. Obes. Rev..

[29] Rothman K.J. (2008). BMI-Related Errors in the Measurement of Obesity. Int. J. Obes..

[30] Pinnick K.E., Nicholson G., Manolopoulos K.N., McQuaid S.E., Valet P., Frayn K.N., Denton N., Min J.L., Zondervan K.T., Fleckner J. (2014). Distinct Developmental Profile of Lower-Body Adipose Tissue Defines Resistance against Obesity-Associated Metabolic Complications. Diabetes.

[31] Sonaglioni A., Ferrulli A., Nicolosi G.L., Lombardo M., Luzi L. (2024). The Influence of Anthropometrics on Cardiac Mechanics in Healthy Women with Opposite Obesity Phenotypes (Android vs. Gynoid). Cureus.

[32] Baigent C., Blackwell L., Emberson J., Holland L.E., Reith C., Bhala N., Peto R., Barnes E.H., Keech A., Cholesterol Treatment Trialists’ Collaboration (2010). Efficacy and Safety of More Intensive Lowering of LDL Cholesterol: A Meta-Analysis of Data from 170,000 Participants in 26 Randomised Trials. Lancet.

[33] Silverman M.G., Ference B.A., Im K., Wiviott S.D., Giugliano R.P., Grundy S.M., Braunwald E., Sabatine M.S. (2016). Association between Lowering LDL-C and Cardiovascular Risk Reduction among Different Therapeutic Interventions: A Systematic Review and Meta-Analysis. JAMA.

[34] Blumenthal R.S., Morris P.B., Gaudino M., Johnson H.M., Anderson T.S., Bittner V.A., Blankstein R., Brewer L.P.C., Cho L., de Ferranti S.D. (2026). 2026 ACC/AHA/AACVPR/ABC/ACPM/ADA/AGS/APhA/ASPC/NLA/PCNA Guideline on the Management of Dyslipidemia: A Report of the American College of Cardiology/American Heart Association Joint Committee on Clinical Practice Guidelines. Circulation.

